# Unlocking Superior Photodetection Properties of Electrodeposited MoS_2_ Quantum Dots

**DOI:** 10.1002/smll.202408001

**Published:** 2025-07-24

**Authors:** Abderrahim Bayou, Bouchra Asbani, Nitul Rajput, Driss Mouloua, Andrea Campos, Abdelilah Lahmar, Khalid Hoummada, Hamid Ouaghaddou, Xiao Zhang, Mimoun El Marssi, Mustapha Jouiad

**Affiliations:** ^1^ Laboratory of Physics of Condensed Matter University of Picardie Jules Verne Scientific Pole, 33 Rue Saint‐Leu, CEDEX 1 Amiens 80039 France; ^2^ Advanced Materials Research Center Technology Innovation Institute P.O. Box 9639 Abu Dhabi 23205 UAE; ^3^ CNRS CEA‐LETI, MINATEC Grenoble INP LTM Université Grenoble Alpes Grenoble F‐38054 France; ^4^ CNRS IM2NP Aix Marseille Université Marseille 13397 France; ^5^ CNRS Institut des Sciences Moléculaires d'Orsay Université Paris‐Saclay Orsay France; ^6^ Département de physique CY. Cergy Paris Université Cergy‐Pontoise Cedex F‐95031 France

**Keywords:** detectivity, electrodeposition, MoS_2_ quantum dots, photodetection, quantum efficiency, responsivity

## Abstract

Quantum dots (QDs) based on transition metal dichalcogenides such as MoS_2_ offer an alternative strategy to yield excellent optoelectronic properties and promote their photodetection performances. By synthesizing MoS_2_ QDs through a controlled electrodeposition process, their superior photodetection properties are unlocked, surpassing those reported in existing literature. Through comprehensive characterization and analysis, the successful fabrication of MoS_2_ QDs made of a mixture of metallic 1T‐MoS_2_ and semiconductor 1T/2H‐MoS_2_ is demonstrated. The photodetection response of MoS_2_ QDs samples shows a synergistic effect between the two nuances of MoS_2_, achieving under a standard solar simulator, 758 A W^−1^ and 5.2·10^13^ Jones for the responsivity and the detectivity. Surprisingly, under UV excitation at 5 V bias for smaller MoS_2_ QDs, the device demonstrates impressive responsivity and detectivity reaching up to 1708.7 A W^−1^ and 1.2·10^14^ Jones, respectively as well as excellent external and internal quantum efficiencies of 5.4·10^5^% and 7.8·10^5^% establishing it as one of the highest‐performing MoS_2_‐based photodetectors reported so far. This research not only sheds light on the potential of MoS_2_ QDs but also paves the way for their integration into photodetection technologies with unprecedented sensitivity.

## Graphical abstract

Broadband MoS_2_ quantum dot photodetectors, composed of mixed‐phase 1T/2H‐MoS_2_ synthesized via a simple pulsed electrodeposition process, exhibit remarkable responsivity and detectivity, along with rapid response and recovery times.

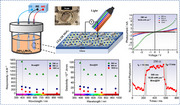

## 1 Introduction

Photodetection in 2D materials is a rapidly growing area of research with promising applications across various fields.^[^ ^1^, ^2^ ^]^ 2D materials, such as graphene and transition metal dichalcogenides like molybdenum disulfide (MoS_2_), possess tunable electronic and optical properties.^[^ ^3^, ^4^, ^5^, ^6^ ^]^ These properties make them highly sensitive to light across a broad spectrum, ranging from UV to infrared wavelengths.^[^ ^3^, ^7^ ^]^ In photodetection, these materials are utilized to convert light signals into electrical signals with exceptional efficiency and sensitivity.^[^ ^2^, ^8^ ^]^ Moreover, their tunable bandgap, high surface‐to‐volume ratio, and high electron mobility offer opportunities to develop compact, lightweight, and low‐power photodetection devices.^[^ ^9^ ^]^ There is still room for exploring novel fabrication techniques and device architectures to unlock the full potential of 2D materials for next‐generation photodetection technologies.^[^ ^10^ ^]^ One promising route is the use of 2D materials as quantum dots (QDs) with controlled size and properties to benefit from their quantum confinement effects. Like graphene, MoS_2_ is isolated by mechanical or chemical exfoliation^[^ ^11^, ^12^, ^13^ ^]^ or fabricated by chemical vapor deposition (CVD).^[^ ^8^, ^14^ ^]^

On the other hand, QDs are nanoscale particles exhibiting discrete optical absorption depending on their size and shape due to their quantum confinement.^[^ ^15^, ^16^ ^]^ Hence, working with QDs made of MoS_2_ would be the key to unlocking its superior photodetection properties, which will open the door to numerous potential applications, particularly in optoelectronics, photovoltaics, sensing, and quantum computing.^[^ ^17^, ^18^ ^]^ However, preparing MoS_2_ QDs using current fabrication methods presents several challenges, including size control, scalability, defects, contamination and purity, as well as reproducibility and yield. To address these challenges, the electrodeposition technique offers a possibility to circumvent these issues for producing MoS_2_ QDs, making it a promising approach in the field. First, electrodeposition provides precise control over the growth process, enabling tunable parameters such as deposition time, voltage, and electrolyte composition to dictate the size, morphology, and density of the QDs.^[^ ^19^ ^]^ Various methods have been reported to produce MoS_2_ films and QDs,^[^ ^13^, ^20^, ^21^, ^22^, ^23^ ^]^ however, the electrochemical deposition technique stands out particularly effective method for fabricating MoS₂ on conducting substrates due to its easy experimental setup requirements, low cost, fast processing, and high‐throughput. Electrodeposition is well‐suited for creating complex structures or micro‐ and nano‐scale features for advanced devices fabrication. Numerous studies have explored the electrodeposition of MoS_2_. However, the majority of the reported studies have primarily focused on conventional electrodeposition techniques using aqueous electrolytes with [MoS₄]^2^⁻ ions as the precursor.^[^ ^24^, ^25^, ^26^, ^27^ ^]^ The most common approach involves the cathodic reduction of an aqueous solution of ammonium tetrathiomolybdate ((NH₄)₂MoS₄).^[^ ^28^, ^29^, ^30^ ^]^ Reports indicate that MoS_2_ thin films prepared via this method are often X‐ray amorphous, consisting of an amorphous matrix embedded with quantum‐sized nanocrystallites or clusters.^[^ ^31^ ^]^ It is only after high‐temperature annealing that these MoS_2_ films become crystalline, with a high degree of texturing, where the Van Der Waals planes are oriented parallel to the substrate. In other study, MoS_2_ thin films have been obtained through oxidative electrodeposition from aqueous solutions containing tetrathiomolybdate ((NH_4_)_2_MoS_4_). The thickness of the resulting films varies with deposition time, but the MoS_2_ remains amorphous.^[^ ^32^ ^]^ Further research demonstrated successful deposition of polycrystalline 2H‐MoS_2_ phase, consisting of a uniform and continuous layered film.^[^ ^33^ ^]^

All these studies involve conventional electrodeposition methods, which are static and do not allow for the fine‐tuning of various electrodeposition parameters, necessary to control the microstructures and the morphologies of the deposits. These methods often lead to ion depletion at the electrode surface, adversely affecting the deposition rate and the quality. Our proposed pulsed electrodeposition technique could potentially address some limitations of traditional methods, such as improving control over deposition parameters and mitigating ion depletion issues, thereby enabling more precise fabrication of MoS_2_ QDs and advanced photodetection devices. Furthermore, electrodeposition can be conducted at relatively mild conditions, reducing the risk of introducing defects or impurities into the MoS_2_ lattice compared to high‐temperature processes like CVD.^[^ ^34^, ^35^ ^]^ Additionally, electrodeposition can be easily scaled up for large‐area deposition, offering potential for high‐throughput production of MoS_2_ QDs suitable for industrial applications.

In this study, we explore the use of the electrodeposition technique to fabricate outstanding MoS_2_ QDs photodetectors, surpassing the conventional reported ones in terms of sensitivity and efficiency. To validate our approach, we provide comprehensive characterizations and analyses supporting our findings, as well as photodetection measurements using a standard solar simulator and various wavelength excitations.

## 2 Experimental Section

### 2.1 MoS_2_ QDs Synthesis

The electrodeposition experiments were carried out using an electrolyte prepared using 10 mm ammonium tetrathiomolybdate and 0.2 m of potassium chloride, mixed in 40 mL of distilled water. During the electrodeposition process, the pH of the electrolyte was adjusted to 7.2, following the method described elsewhere.^[^ ^28^, ^36^ ^]^

**Figure 1:**
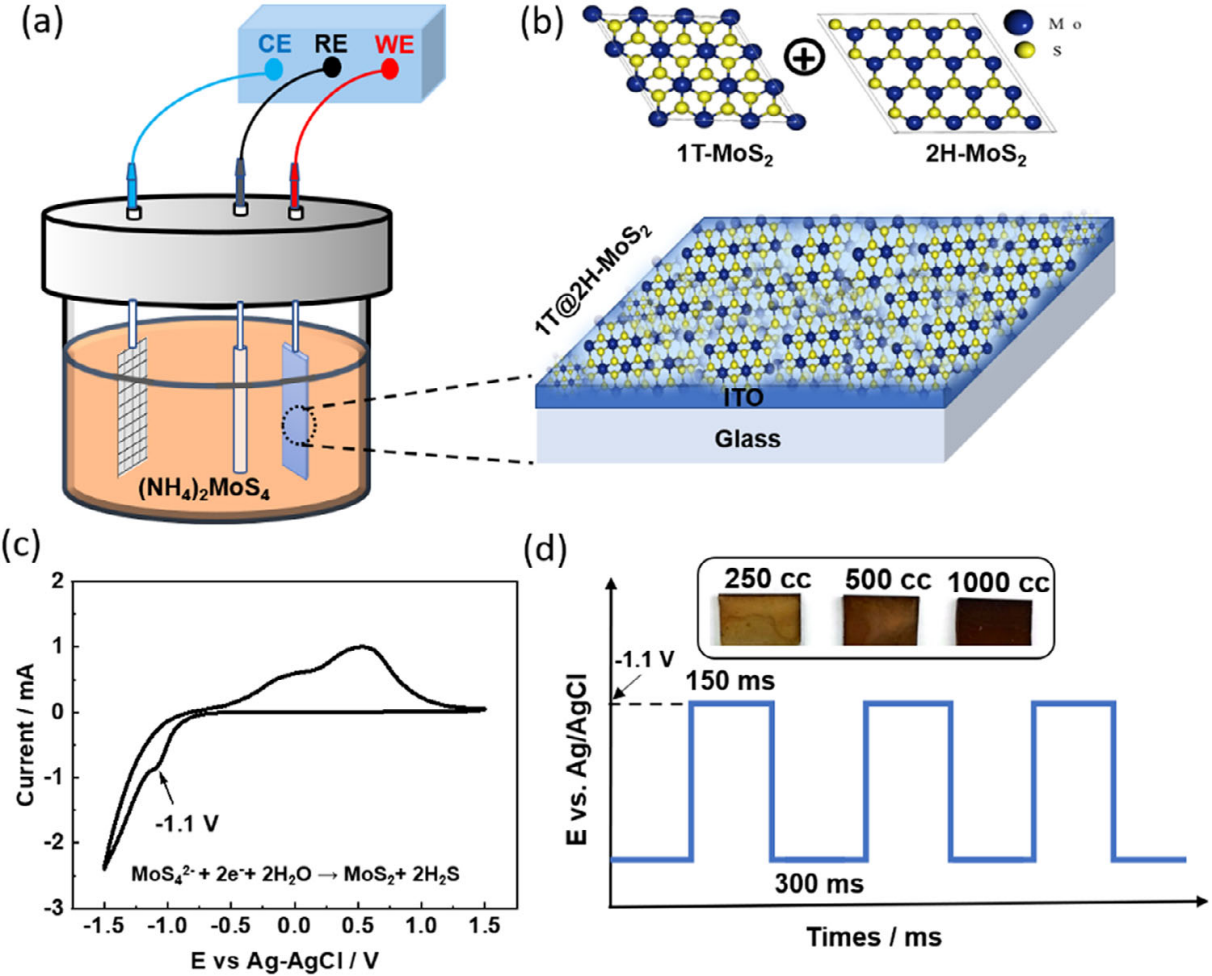
Fabrication protocol of our MoS_2_ QDs. a) Set up of a 3‐electrodes electrochemical cell. b) Electrodeposited MoS_2_ QDs on ITO‐coated glass. c) Cyclic voltammetry curve showing the favorable electrodeposition voltage (−1.1 V vs Ag/AgCl). d) Pulsed electrodeposition profile with ON/OFF cycles at −1.1 V, used to fabricate MSQD_250_ (250 cycles), MSQD_500_ (500 cycles), and MSQD_1000_ (1000 cycles); inset: optical images of the resulting samples.

The electrolyte was selected to allow MoS_2_ deposition onto the Indium Tin Oxide (ITO) coated glass substrate (Sigma–Aldrich). Prior to electrodeposition, the ITO substrates were rinsed successively with distilled water, ethanol, and acetone under sonication for 10 min each. To ensure the electrolyte homogeneity and complete (NH_4_)_2_MoS_4_ dissolution in distilled water, the solution was mixed continuously for 24 h at room temperature using a magnetic stirrer set at ≈230 rpm. Then, the electrodeposition process was carried out in a three‐electrode setup, as shown in **Figure** **1**a, where the ITO substrate was the working electrode, the Platinum mesh served as the counter electrode, and Ag/AgCl (3.5 m KCl) as the reference electrode. First, cyclic voltammetry was performed to determine the deposition (Figure 1b) condition corresponding to the MoS_4_^2−^ reduction, which was found to be −1.1 V versus Ag/AgCl reference electrode, as shown in the cathodic peak potential (Figure 1c). MoS_2_ QDs were deposited on an ITO substrate using a pulsed electrodeposition technique. At an applied potential of −1.1 V versus Ag/AgCl reference electrode, MoS₄^2^⁻ ions in the precursor solution were reduced to MoS_2_ on the electrically conducting ITO electrode. The deposition was performed using an electrolytic solution based on (NH_4_)_2_MoS_4_ with the following procedure: an initial step carried out at −1.1 V versus Ag/AgCl for 0.15 s, followed by a second step at open circuit potential for 0.3 s. The number of deposition pulses controls the thickness of the MoS_2_ QDs. In this study, three conditions were employed: 250, 500, and 1000 cycles, resulting in samples denoted as MSQD250, MSQD500, and MSQD1000, respectively (Figure 1d). This rapid deposition procedure facilitates the formation of a conformal and homogeneous MoS₂ film in a short period of time, and the deposits exhibit good adhesion to the ITO substrate. The use of replicates was essential for accounting for variability in the deposition process and in ensuring that the observed deposits were consistent across different samples. For each deposition time, at least five replicate samples were produced. All samples were prepared under identical conditions to minimize variability, and the results from each replicate were averaged to provide a more accurate representation of the data.

### 2.2 MoS_2_ QDs Characterization

The X‐ray diffraction (XRD) experiments were conducted using a high‐resolution Bruker Discover D8 diffractometer equipped with Cu Kα radiation (λ = 1.5418 Å). The instrument operated at 40 kV and 40 mA and utilized a copper anticathode and a Göbel mirror to produce a diffracted parallel beam. A LynxEye detector in 1D mode was employed to enhance measurement times and improve counting statistics. For the analysis, a grazing incidence mode (GIXRD) was utilized, with a 2θ scan range of 10° to 77°, an incident angle of 0.2°, a step size of 0.02°, and a dwell time of 10 s per step. Raman spectroscopy was performed using a micro‐Raman spectrometer (Raman inVia spectrometer, Renishaw) with a green laser excitation at 532 nm focused by a ×50 objective lens. In situ heating Raman spectroscopy was conducted using a heating stage Linkam Scientific Instruments (Model HFS600E‐PB4, UK). Photoluminescence measurements were performed using Horiba Labram HR evolution equipment with a laser excitation at 532 nm and an InGaAs detector. X‐ray photoelectron spectroscopy (XPS) analyses were carried out utilizing a ThermoFisher Scientific K‐alpha spectrometer and a PHI VersaProbe III scanning XPS microprobe equipped with a 15 kV electron gun generating monochromatic and micro‐focused Al K‐alpha X‐ray source of 1486.6 eV. All XPS spectra were initially calibrated using the C1s peak position reference at 284.6 eV, and the peak fittings were performed using the CasaXPS program. Optical properties were investigated using a UV–vis‐near IR spectrometer, JASCO V‐670, equipped with a 60 mm integrating sphere. The examination of sample structures was performed using a Zeiss (Oberkochen, Germany) Gemini 500 ultrahigh‐resolution field emission scanning electron microscope (FESEM) operating at low voltage (0.3 kV), equipped with an in‐lens detector. Microstructure analysis was further investigated using high‐resolution transmission electron microscopy (TEM) with a Cs‐corrected Titan (ThermoFisher Scientific) operating at 300 kV and electron energy loss spectroscopy (EELS) from Ametek. All TEM lamellas were prepared using the standard FIB lift‐out technique with a dual‐focused ion beam system, Helios (ThermoFisher Scientific). The morphology of the samples was characterized by low‐temperature scanning tunneling microscopy (STM), operated at 77 K. The STM analysis was recorded at constant current mode. The tungsten tip was prepared by electrochemical etching. The photoelectrical measurements were conducted in ambient conditions using an electrochemical workstation potentiostat (VSP‐3e) from BioLogic, monitored by EC‐LAB software. The measurements were coupled with a solar simulator from Ossila controlled by a solar simulator console either under wavelength excitations (390, 459, 515, 630, 730, and 950 nm) or under standard solar light excitation of 1.5AMG at various power densities (40, 60, 80, and 100 mW cm^−2^). The active area of the device for photodetection measurement was ≈5·10^−4^ cm^2^. The device's Pt electrodes were deposited using magnetron sputtering. The schematic of the device structure, along with the inset of the device digital photograph, is shown in Figure S10 (Supporting Information).

## 3 Results and Discussion

### 3.1 MoS_2_ QDs Analysis

**Figure 2:**
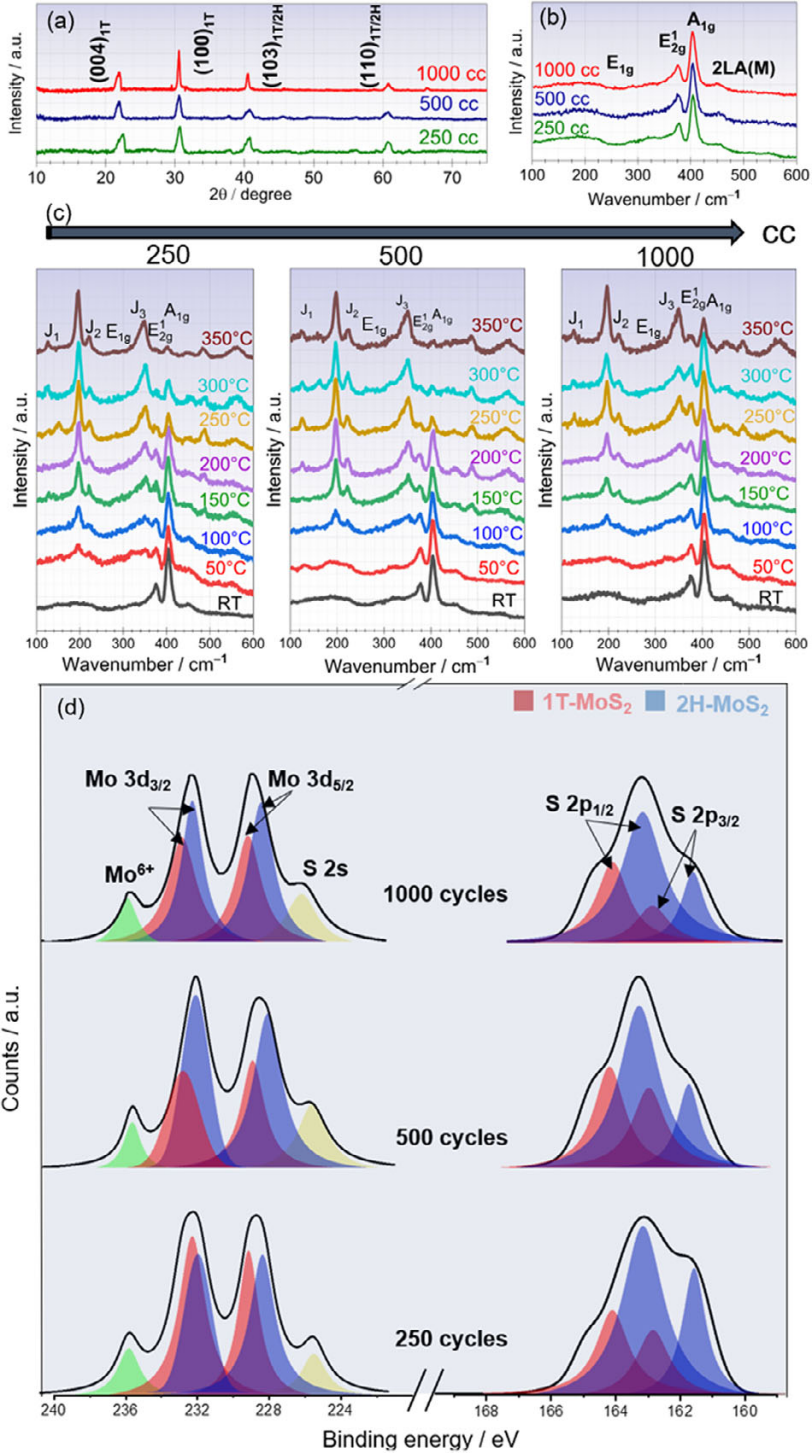
MoS_2_ QDs structural and composition analyses. a) GIXRD diagram showing the crystalline quality of the samples. Mixed phase 1T/2H MoS_2_ is present, and there is a peak shift toward lower angles with increasing deposition cycles. b) Raman peaks showing the vibrational modes of the mixed phase 1T/2H‐MoS_2_, with a broad peak at 200 cm⁻¹ highlighting the 1T‐MoS_2_ vibrational modes. c) In situ Raman heating (from RT to 350 °C) showing the evolving 1T‐MoS_2_ characteristic Raman peaks. It is observed that the vanishing of 2H‐MoS_2_ characteristic Raman peaks. d) XPS spectra of all samples, the deconvolution of Mo 3d and S 2S core levels indicates the presence of mixed phase 1T/2H‐MoS_2_.

**Figure** **2**a illustrates the grazing incident X‐ray diffraction (GIXRD) diagram of our MoS_2_ samples. We observe the presence of typical diffraction peaks of both 2H‐MoS_2_ and 1T‐MoS_2_ mixture phases at 30.8, 40.8°, and 60.6° corresponding to (100), (103), and (110) planes, respectively.^[^ ^37^, ^38^ ^]^ Notably, an extra characteristic peak 1T‐MoS_2_ at ≈21.5° attributed to (004) plane is observed for all samples.^[^ ^39^ ^]^ Besides, a peak shift toward low angles with increasing deposition cycles (from MSQD_250_ toward MSQD_1000_) is visible. This shift is more pronounced for the (004)_1T_ peak. We believe this shift may occur for several reasons, including the transformation of MoS_2_ from 2H phase to 1T phase, which is often associated with an increase in interlayer spacing. Additionally, other researchers have noted that the accumulation of strain and defects during multiple deposition cycles can further modify the lattice parameters, contributing to the observed peak shift.^[^ ^40^, ^41^ ^]^

Figure 2b depicts the Raman peaks for all samples. The typical vibrational modes of 2H‐MoS_2_, are designated by E_1g_ ≈296 cm^−1^, E^1^_2g_ ≈378 cm^−1^ and A_1g_ ≈404 cm^−1^ peaks.^[^ ^42^, ^43^ ^]^ Interestingly, at lower vibration frequencies, we observe a convoluted spectrum. Upon fitting this spectrum, we identify cumulative J_1_, J_2_, and J_3_ peaks, corresponding to 1T‐MoS_2_ as well,^[^ ^43^, ^44^ ^]^ the Raman peaks shows the vibrational modes for both 1T/2H‐MoS_2_ phases, with the insert showing the deconvolution peaks highlighting the 1T‐MoS2 vibrational modes (Figure S1, Supporting Information). which is consistent with the GIXRD results. Combining both findings, all MoS_2_ QDs samples exhibit a mixed‐1T/2H‐MoS_2_ phase as reported elsewhere.^[^ ^45^, ^46^ ^]^ Similar to XRD, Raman peaks are known to be highly sensitive to strain variation. The deposition of subsequent MoS_2_ layers can soften the phonon modes, leading to peak shifts to lower frequencies. Additionally, the increased interlayer interactions and their coupling as more layers are deposited may also contribute to the frequency shift.^[^ ^47^ ^]^

The evolving crystal structure with temperature is further examined using in situ heating Raman spectroscopy. The Raman peaks of the samples heated from 30 to 350 °C, are depicted in Figure 2c. We notice the typical Raman peaks 1T‐MoS_2_ phase at J_1_ (147 cm^−1^), J_2_ (223 cm^−1^), and J_3_ (346 cm^−1^)^[^ ^48^, ^49^ ^]^ become gradually sharper and more defined with increasing temperature, while those corresponding to 2H‐MoS_2_ phase at E_1g_ (296 cm^−1^), E^1^_2g_ (378 cm^−1^) and A_1g_ (404 cm^−1^), and 2LA(M) (450 cm^−1^) are gradually vanishing.^[^ ^49^ ^]^ This finding confirms the coexistence of 1T/2H‐MoS_2_ mixed phases at ambient and the disruption of this mixture with increasing temperature. It is clear that 1T/2H‐MoS_2_ mixed phases are dominated by the semiconducting 2H‐MoS_2_ at low temperature, whereas the metal 1T‐MoS_2_ phase prevails at high temperatures.^[^ ^46^, ^49^, ^50^ ^]^ Additionally, we observe the complete degradation of 2H‐MoS_2_ at 350 °C for MSQD_250_ and MSQD_500_ samples. Substantial, in situ Raman heating investigation at various temperatures, including the influence of dwell time conducted on all samples, is provided in Figures S2–S4 (Supporting Information). The obtained results concur with the presence of 1T/2H‐MoS_2_ mixed phases, where 2H‐MoS_2_ is the prevailing phase at room temperature, and the 1T‐MoS_2_ phase dominates whether at higher temperatures or at lower temperatures, but at prolonged dwell times. It is important to note that the metastable phase pertains to 1T'‐MoS_2_ is challenging to stabilize.^[^ ^51^ ^]^ However, this study concerns the 1T‐MoS_2_ phase, which can be readily stabilized in the presence of the 2H‐MoS_2_ phase.^[^ ^44^ ^]^ As noted in their review article, the metastable T‐MoS_2_ phase is stabilized by interacting with the 2H‐MoS_2_ phase, particularly when they form a heterostructure. In our study, we also observe a heterostructure comprising both 1T‐MoS_2_ and 2H‐MoS_2_ phases as demonstrated by HRTEM images. Additionally, we have reported in previous work that the 1T‐MoS_2_ can be stabilized in the presence of 2H‐MoS_2_ at high temperature using CVD deposition.^[^ ^38^ ^]^ We believe that the clustering of Mo atoms and the resulting structural distortion could contribute to the stabilization of 1T‐MoS_2_, provided there is a favorable orientation of a stable phase, such as the 2H‐MoS_2_ phase nearby. Other studies have also explored the stability of 1T‐MoS_2_ alone using different fabrication methods.^[^ ^52^, ^53^, ^54^ ^]^

**Table 1:** Comparison of our MSQD_250_ with reported studies using different laser excitations. t_r_: rise time; t_d_: decay time; MSM: Metal‐semiconductor‐Metal; NPs: Nanoparticles; BP: Black Phosphorus.

Materials	Route	Bias [V]	λ [nm]	*P*_ligh_ [mW cm^−2^]	R [A W^−1^]	D^*^[Jones]	EQE [%]	t_r_/t_d_	Refs.
MSQD_250_	Electro‐deposition	5	390	16.85	1708.7	1.2·10^14^	5.4·10^5^	44/51ms	This work
MoS_2_‐MSM	Thermolysis	10	532	–	0.57	10^10^	1.3·10^2^	70/110µs	[61]
MoS_2_/Si	Thermolysis	−2	650	90	11.9	2.1·10^10^	2.2·10^3^	30.5/71.6µs	[71]
MoS_2_	PLD	10	445	8	0.05	1.5·10^9^	–	–	[62]
MoS_2_‐MSM	Thermolysis	5	532	2	1.04	–	2.4·10^2^	40/50µs	[72]
MoS_2_‐QDs/Si	Exfoliation	−2	514	–	1	8·10^11^	2·10^2^	1.5/2.5ns	[63]
MoS_2_‐MSM	CVD	3	532	100	0.55	–	–	0.2/1.7ms	[64]
MoS_2_/MoTe_2_	Exfoliation	–	532	100	0.62	10^10^	144	< 10µs	[73]
MoS_2_/GaN	CVD	20	460	–	0.25	5.6·10^8^	67.4	2.2/4.4s	[74]
MoS2/p‐Si	Sputtering	0	455	5.2	0.03	1.4·10^10^	–	38.8 /43µs	[75]
MoS2/Au‐NPs	Hydrothermal	2	554 780	0.25	0.09 0.06	3.5·10^10^ 2.2·10^10^	22.23 9.71	0.9/−s 1/−s	[65]
MoS_2_/BaTiO_3_‐QDs	Spin‐Coating	1	365	0.1	120	1.1·10^11^	4.8·10^4^	0.7/0.2s	[76]
MoO_x_/MoS_2_	CVD	10	405	1	1.09	2.1·10^11^	–	9.8 /12.6s	[77]
MoS_2_/BP	Exfoliation	3	532	–	22.30	3.1·10^11^	10^2^	–	[78]
MoS_2_/Al_2_O_3_ MoS_2_/HfO_2_	Exfoliation	5 5	635 635	3.5 41	406 434	3.8·10^11^ 7.7·10^11^	– –	− /55 ms − /7ms	[79]
GaSe/MoS_2_	Sputtering	1	520	–	0.67	2.3·10^11^	1.6·10^2^	155/215ms	[70]

Furthermore, we utilize XPS to examine the Mo 3d and S 2p core levels of our samples as illustrated in the high‐resolution XPS spectra of Figure 2f. The XPS spectra exhibit broad peaks for both Mo 3d and S 2P core levels, which are relatively similar for all samples. Further analysis of the XPS spectra shows that the Mo 3d orbital displays six distinct components, namely Mo^6+^, Mo 3d_3/2_, Mo 3d_5/2,_ and S 2s peaks, confirming once again the mixed phases 1T/2H‐MoS_2_.^[^ ^38^, ^55^ ^]^ Additionally, the presence of the S‐2s peak underscores the excellent crystalline quality of the MoS_2_ structure,^[^ ^56^, ^57^ ^]^ while the presence of the Mo^6+^ peak indicates the existence of Mo states within the surface of oxides.^[^ ^58^ ^]^ Similarly, the deconvolution of the high‐resolution S 2p peak also suggests the coexistence of 1T/2H‐MoS_2_ mixed phases, evidenced by the distinct S 2p_1/2_ and S 2p_3/2_ peaks. Furthermore, we observe an increase in the proportion of the 1T‐MoS_2_ phase with an increasing number of deposition cycles. This is due to the cumulative effects of strain, defects, and changes in the growth conditions that favor the stabilization of the 1T‐MoS_2_ phase. As more layers are deposited, the increasing strain and defect density disrupt the equilibrium, facilitating the phase transition from the 2H‐MoS_2_ phase to 1T‐MoS_2_ phase. Using XPS data, we determined the ratio of 1T‐MoS_2_ with respect to 2H‐MoS_2_ from the Mo3d 3/2 peak (Table S1, Supporting Information). The atomic percentage of each component was calculated using the CasaXPS software. Similarly, the calculated Mo/S ratios for all deposited samples are shown in Table S2 (Supporting Information), our results show that all deposits are S‐rich, especially the MoS2QD_500_ and MoS2QD_1000_ samples.

**Figure 3:**
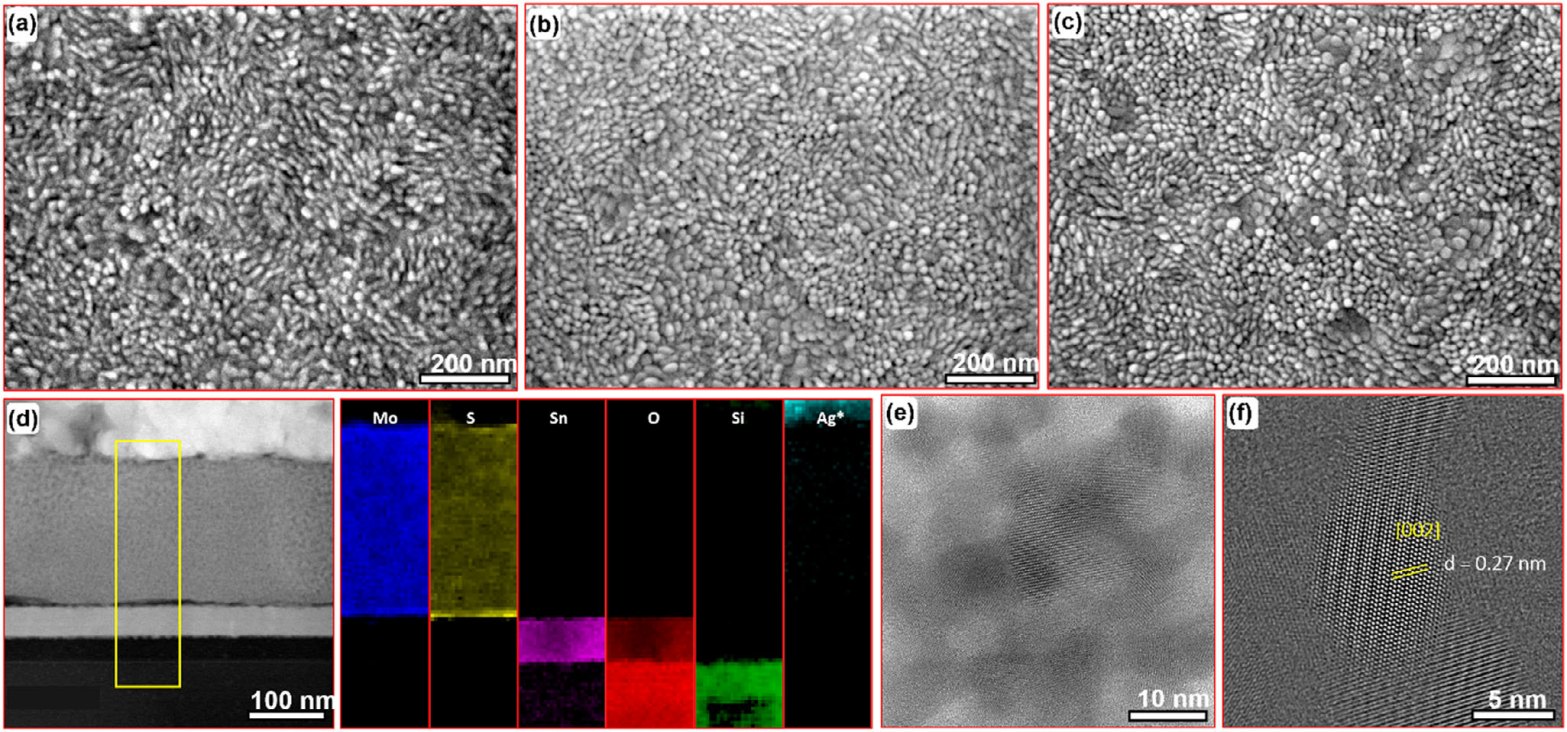
MoS_2_ QDS microstructural analysis. Typical high‐magnification SEM micrographs of electrodeposited samples: a) MSQD_250_, b) MSQD_500_, and c) MSQD_1000_. d) Cross‐sectional TEM image of MSQD_500_ and the corresponding EELS mapping of the yellow rectangle showing Mo, S (sample), Sn, O, Si (ITO coated glass substrate), and Ag is the capping layer. HRTEM images of MSQD_500_ showing well‐crystalline QDs of a few nanometers. e) Large view. f) Zoom‐in on two QDS.

Hereafter, we examined the microstructural and morphological structures of our samples. The resulting high‐magnification SEM images are presented in **Figure** **3**a–c, suggesting smooth and continuous deposition of the MoS_2_ QDs, exhibiting a granular shape. SEM images at low magnification are provided in Figure S5 (Supporting Information). The QDs size appears to vary with the deposition cycles, measuring in average ≈3.5, ≈12.6, and ≈28.4 nm for samples MSQD_250_, MSQD_500_, and MSQD_1000_, respectively (TEM results for MSQD_250_, STM images recorded for MSQD_500_, and size distribution for all samples are shown in Figure S6–S8, Supporting Information, respectively). As observed, the QDs size increases with increasing number of deposition cycles, which can be attributed to the nucleation and growth processes during the electrodeposition. In the initial cycles, small nuclei of MoS₂ form dendritically on the substrate. As more deposition cycles are carried out, these nuclei grow and coalesce due to the continuous deposition of Mo and S atoms, resulting in larger particle sizes as reported elsewhere.^[^ ^59^ ^]^

Subsequently, we prepared a cross‐sectional TEM lamella and STEM‐EELS mapping is provided in Figure 3d. The image represents the high‐angle annular dark field STEM image of a cross‐sectional view of MSQD_500_ ≈240 nm thick, on top of an indium tin oxide (ITO) substrate. The EELS mapped elements are Mo, S, Sn, O, Si and Ag (coating layer). The MoS_2_ region has Mo and S as expected, Sn indicates the ITO coating on glass represented by Si and O. Figure 3e illustrates the HRTEM image showing MSQD_500_ embedded in an amorphous matrix. A close view allows to capture of two crystals interconnected along [112]_H_ // [011]_T_ directions (Figure 3f), consisting of MoS_2_ nanocrystals (≈10 nm) of mixed phases of 2H‐MoS_2_ represented by [002] zone axis and the 1T‐MoS_2_ indicated by d_011_ direction, confirming the above findings.

### 3.2 Optical and Photodetection Performances

**Figure 4:**
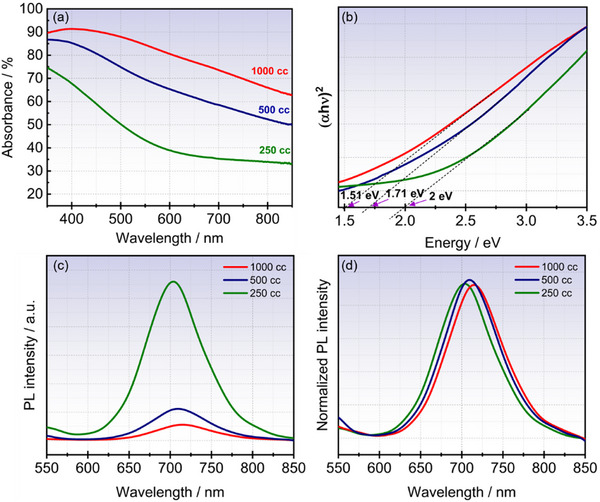
MoS_2_ QDs optical properties. a) UV–vis absorbance: excellent absorbance in the UV region pertaining to the mid visible region. b) Tauc plot and bandgap energy determination, values are indicated in the graph. c) Steady‐state photoluminescence (PL) measurements showing the high absorption of the samples. d) PL close view in the Gaussian spectra showing a slight redshift for high QDs size.

**Figure** **4**a depicts the optical absorption of the QDs samples, exhibiting a broadband optical absorption covering the full 350–850 nm range, and reaching up to 80% for MSQD_1000_, then it decreases with decreasing deposition cycles, reaching 40% for MSQD_250_. The difference in the optical absorption intensities is attributed to sample thickness as demonstrated by cross‐sectional HRTEM images. Using the optical absorption coefficient extracted from the optical transmittance (Figure S9, Supporting Information), we determine the bandgap energy (*E_g_*) for each sample according to Tauc equation:

(1) $${\left(\alpha h\nu \right)}^{2}=A\left(h\nu -E_{g}\right)$$

where *h* is Planck's constant, ν is the photon's frequency, α is the absorption coefficient, and *A* is the slope of the Tauc plot in the linear region (Figure 4b). The selection of the Tauc formula for direct bandgap materials aligns with the strong PL signal observed for our samples (Figure 4c), particularly the sharp and narrow peak in the PL spectra.

We observe a decrease in the bandgap energy with increasing deposition cycles from 2 eV for MSQD_250_ down to 1.51 eV for MSQD_1000_. The decrease in the bandgap energy is confirmed by the redshift observed in the PL spectra for MSQD_500_ (Eg = 1.74 eV) and MSQD_1000_ (Eg = 1.68 eV) (Figure 4d). The shift toward higher wavelengths recorded in PL response confirms the small QDs size of MSQD_250_ (Eg = 1.8 eV) compared to larger sizes for other samples. In other words, smaller QDs exhibit a confinement of charge carriers within a smaller volume, resulting in a higher bandgap energy.^[^ ^60^ ^]^

**Figure 5:**
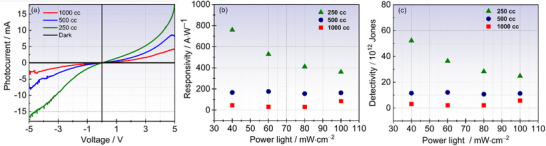
MS_2_ QDs photodetection measurements under solar simulator. a) Photocurrent compared with the dark current (black plot) recorded at P_light_ = 100 mW cm^−2^ and 5 V bias. MSQD_250_ exhibits a very high photocurrent of 18 mA. b) Responsivity as a function of P_light_, suggesting two domains of responsivity for MSQD_250_, and a steady state behavior for other samples. c) Detectivity as a function of P_light_ showing the same trend as for the responsivity.

In the following section, we assess the photodetection capabilities of our as‐deposited MoS_2_ QDs through the measurement of photocurrent (*I_ph_*). **Figure** **5**a showcases the *I–V* curves recorded from our devices under two distinct conditions: in the dark depicted by the black curve, and under solar simulator illumination. The photoresponse plots are generated using a voltage sweep ranging from −5 to + 5 V and employing the following formula:

(2) $$I_{ph}=I_{light}-I_{dark}$$

(3) $$J_{ph}=\frac{I_{ph}}{A}$$

where *I_dark_* and *I_light_* are the currents recorded in darkness and under illumination, respectively, and A denotes the sample active surface area of 5·10^−4^ cm^2^. We measure dark current of 2.1 × 10^−6^, 9.5 × 10^−5^, and 3.3 × 10^−4^ mA and at 1, 3, and 5 V, respectively (Figure S11, Supporting Information). Remarkably, at power density P_light_ of 100 mW cm^−2^ and 5 V bias, MSQD_250_ exhibits a significantly high photocurrent of 18 mA compared to 8.20 mA for MSQD_500_ and 4.5 mA for MSQD_1000_. These excellent photodetection performances, surpassing reported values^[^ ^56^, ^58^, ^61^, ^62^, ^63^, ^64^, ^65^ ^]^ stem the inherent quantum effects present in our sample's optoelectronic behavior. Notably, the superior photodetection response is obtained for the smaller size MSQD_250_. This strong correlation between the film thickness and the size of the QDs was also reported elsewhere.^[^ ^56^, ^63^ ^]^ Additionally, the photoresponse stability and reproducibility tests are carried out on the samples using chronoamperometry experiments. Our findings reveal high stability of the normalized photocurrent over 8 successive cycles (dwell time 20 ms) recorded under standard solar simulator (P_light_ = 100 mW cm^−2^) shown in Figure S12 (Supporting Information). We investigated the stability and the potential photothermal effects on the photoresponse of our devices by measuring their optoelectronic performances across various temperatures. Figure S14 (Supporting Information) (e.g., S.I.) presents the photocurrent measurements for MSQD_250_ electrodeposited at room temperature, 50, 100, and 200 °C. Our measurements show no significant change in photocurrent values with increasing temperature. This suggests that the photothermal effect does not notably impact the photoresponse of our samples.

To further assess the photoconduction properties, we delve into the critical parameters of a photodetector, namely responsivity (R) and detectivity (D^*^) under solar simulator illumination at various P_light_ (40, 60, 80, and 100 mW cm^−2^). R quantifies the conversion efficiency of incident irradiation into electrical current, while D^*^ characterizes the detector ability to detect weak signals. Both R and D^*^ are derived from the following equations:^[^ ^66^, ^67^, ^68^, ^69^ ^]^

(4) $$R=\frac{I_{ph}}{A.P_{\mathrm{light}}}\;$$

(5) $$D^{*}=\frac{R\sqrt{A}}{\sqrt{2qI_{dark}}}$$

where q is the electron charge (1.6·10^−19^C).

The calculated R and D^*^ at 5 V for all samples are depicted in Figure 5b,c, respectively. Both R and D* show a declining trend with increasing incident P_light_. The exceptional photodetection performances of MSQD_250_ are clearly evident for both R and D^*^; MSQD_250_ achieves a remarkably high R of 758 A W^−1^ and D^*^ of 5.2·10^13^ Jones at P_light_ = 40 mW cm^−2^ (Table S2, Supporting Information). Interestingly, both R and D^*^ decrease for higher P_light_, reaching a plateau at P_light_ = 80 mW cm^−2^, indicating average values of 410 A W^−1^ and 2.2·10^13^ Jones for R and D^*^, respectively. Conversely, MSQD_500_ and MSQD_1000_ samples exhibit relatively stable R and D* values across all used P_light_, maintaining steady state values of ≈176 A W^−1^ and 1.2·10^13^ Jones for MSQD_500_ and 85 A W^−1^ and 8.5·10^12^ Jones for MSQD_1000_.

**Figure 6:**
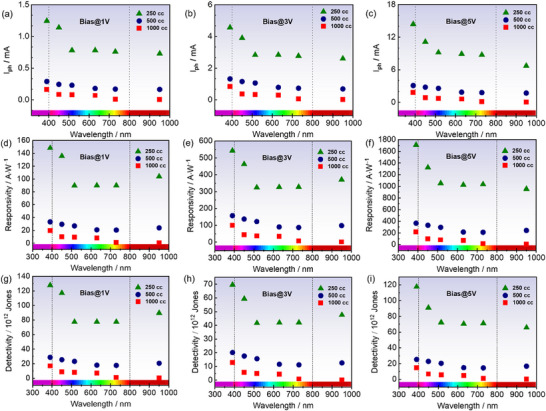
MoS_2_ QDs photodetection properties for all samples as a function of wavelength at 1, 3, and 5 V bias. a–c) Photocurrent. d–f) Responsivity. g–i) Detectivity.

Additionally, in order to explore the photodetection properties of our samples across a spectrum of wavelengths, we conducted analogous measurements as above and substituting the solar simulator with various wavelength excitations spanning from UV to infrared (390–950 nm). For all photodetection measurements, the power density *P*_light_ was set to 16.85 mW cm^−2^ and kept constant across all wavelengths to ensure consistent comparison between samples. The obtained *I_ph_*, R, and D^*^ are plotted against wavelengths at 1, 3, and 5 V bias (**Figure** **6**). Two distinct photodetection domains consistently emerge regardless of the applied bias. The first domain, encompassing the UV region, showcases the highest performance of *I_ph_*, R and D^*^ across all samples, whereas the second domain, covering the visible and IR regions, exhibits comparatively lower performances. Notably, at 5 V bias, our photodetector device fabricated using MSQD_250_, demonstrates impressive *I_ph_*, R, and D^*^ values at 390 nm excitation, reaching up to 14.4 mA, 1708.7 A W^−1^, and 1.2·10^14^ Jones, respectively, followed by a rapid decline reaching a plateau at 515 nm, corresponding to 9.1 mA, 1051.3 A W^−1^, and 7.2·10^13^ Jones, for *I_ph_*, R, and D^*^ respectively. Conversely, MSQD_500_ and MSQD_1000_ show steady state behavior for both R and D^*^ ≈164.1 A W^−1^ and 1.1·10^13^ for MSQD_500_ and 85 A W^−1^ and 5.8·10^12^ for MSQD_1000_. Once again, MSQD_250_ emerges as the superior photodetector among the three considered samples. Using the obtained *R* and D* values, we evaluate the quantum efficiency of our devices. In the following, we delve into the assessment of both external and internal quantum efficiencies (EQE and IQE). The EQE considers the overall efficiency, encompassing both the absorption of light and the collection of generated charge carriers. In contrast, the IQE reflects the efficiency within the device itself, specifically the ratio of absorbed photons that successfully generate electron‐hole pairs within the active zone. Both EQE and IQE are estimated as follows:^[^ ^70^ ^]^

(6) $$\mathrm{EQE}\left(\%\right)=\frac{hc}{q\lambda }R\times 100$$

(7) $$\mathrm{IQE}\left(\%\right)=\frac{EQE\left(\%\right)}{Absorbance\left(\%\right)}\times 100$$

where h is the Planck's constant (6.62 ·10^−34^ J·s), c is the speed of light (3·10^8^ m s^−1^), *R* is the responsivity, *q* is the electron charge (1.6 ·10^−19^C), and λ is the wavelength of incident light.

**Figure 7:**
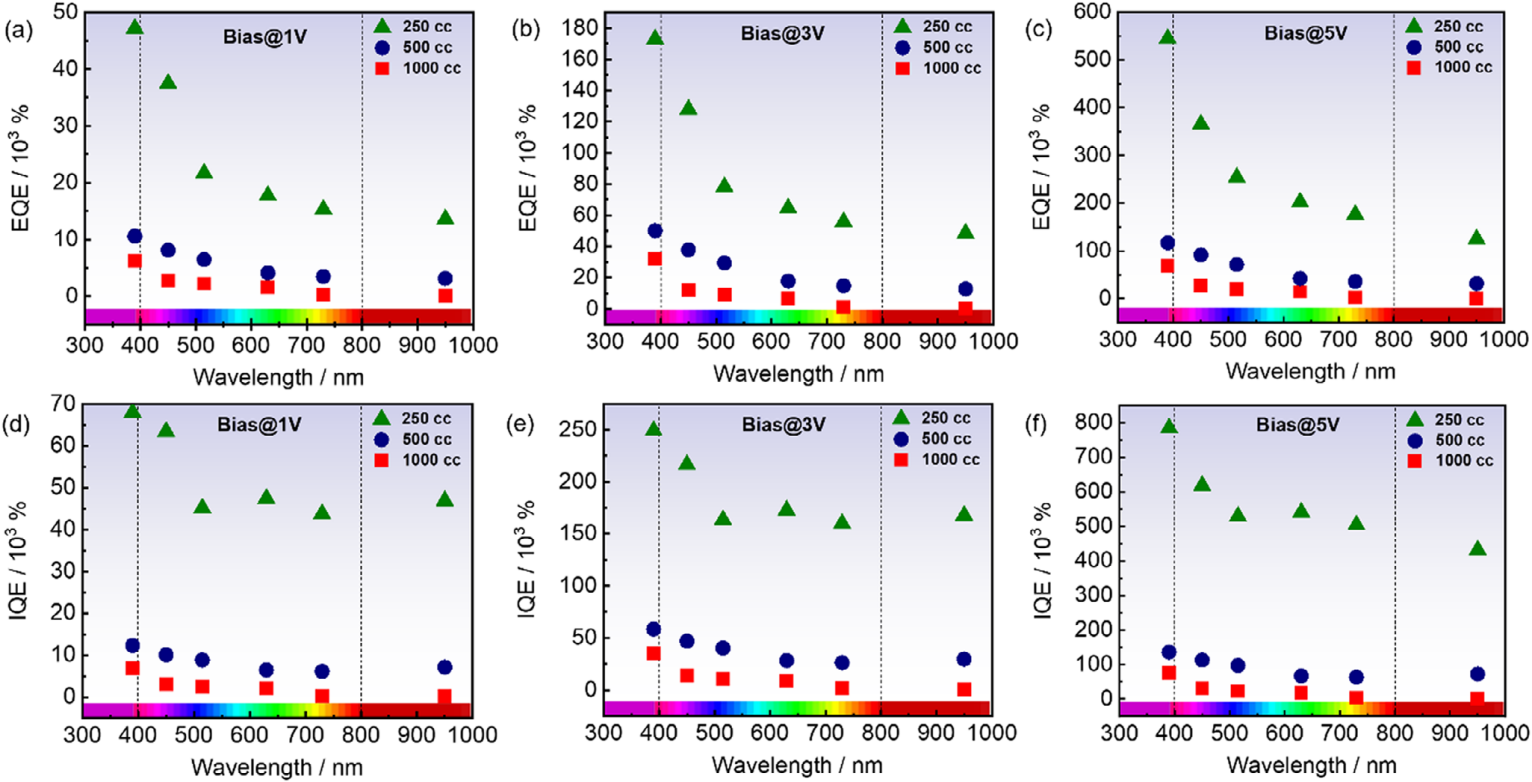
Quantum efficiencies EQE and IQE for all samples as a function of wavelength at 1, 3, and 5 V bias. a–c) EQE showing two domains of behavior for MSQD_250_ and a plateau behavior for other samples. d–f) IQE showing the same trend as the EQE, with the highest values achieved by MSQD_250_ sample.

**Figure** **7** declines the estimated EQE and IQE for all samples across various wavelengths at 1, 3, and 5 V bias. Interestingly, our finding reveals two distinct photodetection regimes for all samples, while it is more pronounced for MSQD_250_. The first domain lies in the UV‐near visible region, specifically from 390 to 500 nm. In particular, the EQE reaches 5 V, a maximum of 5.4·10^5^% under 390 nm excitation for MSQD_250_, indicating a giant number of photons are absorbed. At the same time, the IQE attained a maximum of 7.8·10^5^%, suggesting photoconductive gain, in contrast to avalanche multiplication, where a single absorbed photon generates multiple electron‐hole pairs through a cascading effect.^[^ ^67^ ^]^ The gain obtained arises from photoconductive effects, where the incident light increases the material's conductivity, enhancing the detector's response signal, leading to high IQE values. In the second domain (vis‐near IR), spanning in 500–950 nm range, we obtain a plateau for all samples, corresponding to a steady state behavior of EQE and IQE. The obtained values remain very high and quasi‐stable across the entire considered region, reaching at 5 V bias, average values for MSQD_250_, EQE ≈2·10^5^% and IQE ≈5·10^5^% compared to both MSQD_500_ and MSQD_1000_ attaining, EQE ≈5·10^4^% and IQE ≈8·10^4^%.

To comprehend the mechanism underlying these unprecedented photodetection performances of our MoS_2_ QDs devices, we first benchmark the key markers such as responsivity, detectivity, quantum efficiencies, and sensitivity of our photodetector with reported ones in the literature (**Table** **1**).

The performance metrics under consideration include responsivity, detectivity, and time response, under a specific applied bias as well as the EQE, which are the critical parameters for evaluating photodetector performances. Our MSQD_250_‐based photodetector demonstrates superior performance across all these metrics. Specifically, the responsivity of our device is significantly higher compared to those reported in the literature (10–100 folds). This indicates that our photodetector can generate a stronger electrical signal per unit of optical power, highlighting its enhanced sensitivity to incident light. In terms of detectivity, our photodetector also excels (up to 10^3^‐fold). Higher detectivity values suggest better performance in distinguishing weak signals from noise. This improvement is attributed to synergistic effects between the two phases 1T‐MoS_2_ and 2H‐MoS_2,_ and quantum confinement effect enabled by the small QDs size embedded in an amorphous surrounding. The optimized design of the MSQD_250_ appears to significantly contribute to reduced noise levels and enhanced signal‐to‐noise ratio, as showcased by the high detectivity value. Moreover, the response times of our photodetector, shown in Figure S13 (Supporting Information), are an additional attribute of its excellent performance as it is notably shorter than those in the reported studies. A fast time response is crucial for applications requiring rapid detection and processing of optical signals. Our device's fast response time can be linked to the efficient charge transport mechanisms within the MSQD_250_ material linked to a shorter mean free path due to the size effect and to the signal exaltation by the presence of metallic 1T‐MoS_2_ at the vicinity of 2H‐MoS_2_. Both are enabling swift carrier dynamics and minimal delay. The comparative data in Table 1 underscore the advancements achieved with the MSQD_250_‐based device, promoting our photodetector for a wide range of applications, including high‐speed optical communications, sensitive imaging systems, and advanced sensor technologies. Our result suggests that there is still potential for improvement, particularly in the sieving process of MoS_2_ QDs to achieve uniform particle size. Despite the exceptional photodetection performance of our MoS_2_ QDs, the current measurements represent an average response across all the particles within the excited region of interest in our device. Basically, the region includes a distribution of particle sizes, some of which are larger. We anticipate that MoS_2_ QDs with uniform size, evenly distributed across the substrate, could lead to substantially higher performance.

## 4 Conclusion

We demonstrated the capabilities of MoS_2_ QDs fabricated by controlled electrodeposition technique to exhibit outstanding photodetection performances under both standard solar simulation and under UV excitations. Our processing route enabled the superior photodetection properties of MoS_2_ QDs by favoring a synergistic interaction between metallic 1T‐MoS_2_ and semiconductor 2H‐MoS_2_ at the nanoscale enhanced by the quantum confinement effect. The photodetection response of our best performing MoS_2_ QDs sample has reached up to 1708.7 A W^−1^ and 1.2·10^14^ Jones, respectively, for responsivity and detectivity, under UV excitation at 5 V bias and external and internal quantum efficiencies of 5.4·10^5^% and 7.8·10^5^%, respectively. This excellent performance is promoting our device based on MoS_2_ QDs to a viable and highly sensitive photodetector. Our current work has demonstrated the potential of a controlled electrodeposition technique, which is a facile and scalable method for producing high‐performing photodetectors, paving the way for their integration into photodetection technologies with unparalleled sensitivity and efficiency.

## Conflict of Interest

The authors declare no conflict of interest.

## Supporting information



Supporting Information

## Data Availability

The data that support the findings of this study are available from the corresponding author upon reasonable request.
