# Supplementary material for: Unlocking Superior Photodetection Properties of Electrodeposited MoS2 Quantum Dots
Source: Small. 2025 Jul 24;21(36):e08001. doi: 10.1002/smll.202408001 (PMC12423911; doi:10.1002/smll.202408001)
Supplement: Supplementary file 1 — Supporting Information [file SMLL-21-e08001-s001.docx]

**Supplementary information**

**Unlocking Superior Photodetection Properties of Electrodeposited**

**MoS_2_ Quantum Dots**

Abderrahim Bayou^1^, Bouchra Asbani^1^, Nitul Rajput^2^, Driss Mouloua^3^, Andrea Campos^4^, Abdelilah Lahmar^1^, Khalid Hoummada^4^, Hamid Ouaghaddou^5,6^, Xiao Zhang^5^, Mimoun El Marssi^1^, Mustapha Jouiad^1,*^

^1^Laboratory of Physics of Condensed Matter, University of Picardie Jules Verne, Scientific Pole, 33 Rue Saint-Leu, CEDEX 1, 80039 Amiens, France

^2^Advanced Materials Research Center, Technology Innovation Institute, P.O. Box 9639, Abu Dhabi, United Arab Emirates

^3^Université Grenoble Alpes, CNRS, CEA-LETI, MINATEC, Grenoble INP, LTM, F-38054 Grenoble, France

^4^Aix Marseille Université, CNRS, IM2NP, 13397, Marseille, France

^5^Université Paris-Saclay, CNRS, Institut des Sciences Moléculaires d’Orsay, Orsay, France.

^6^Département de physique, CY. Cergy Paris Université, F-95031 Cergy-Pontoise Cedex, France

^*^Corresponding author: mustapha.jouiad@u-picardie.fr

**
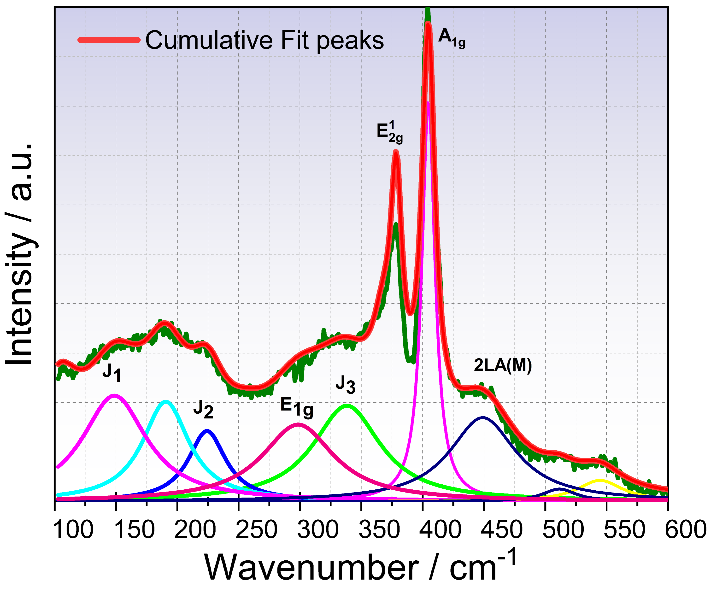
**

**Fig. S1** Raman spectra showing the vibrational modes for mixed phase 1T/2H-MoS_2_. The deconvolution of the broad peak at 200 cm^-1^ highlights the 1T-MoS_2_ phase vibrational modes.

**
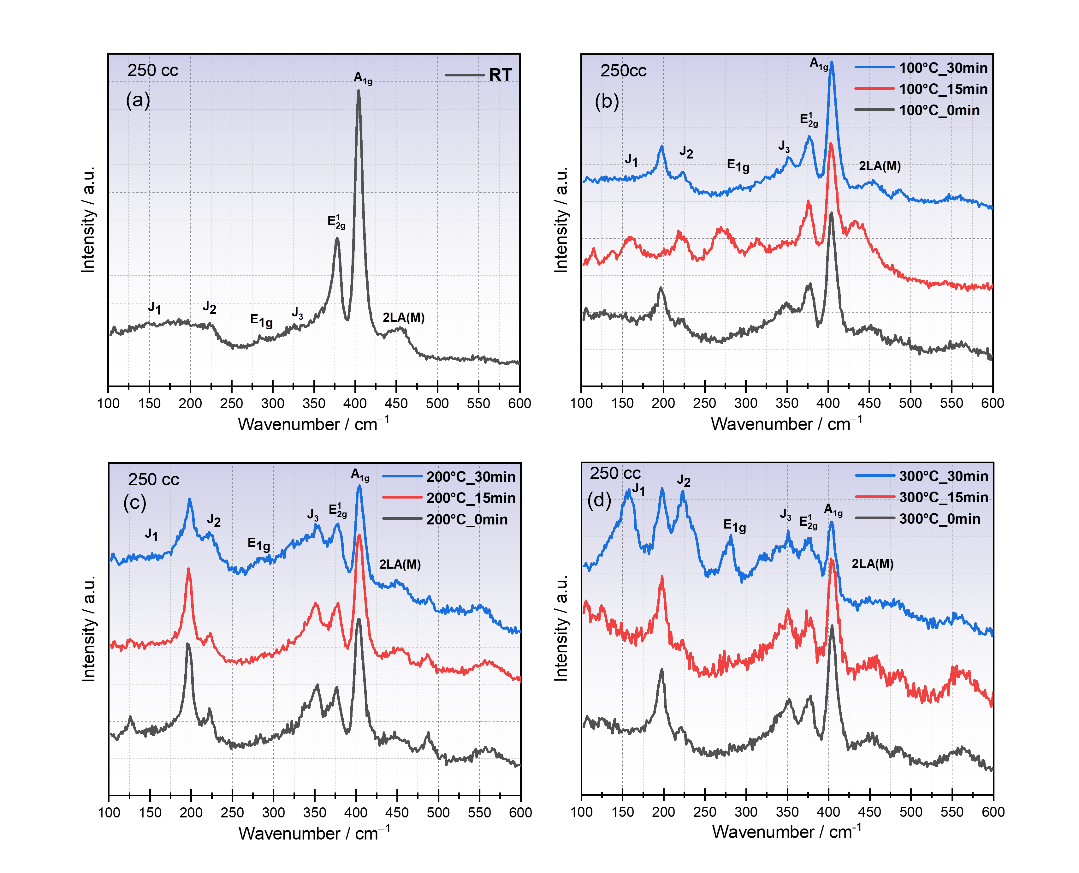
**

**Fig. S2** In situ Raman heating for MSQD_250_ sample recorded at **(a)** RT. **(b)** 100°C. **(c)** 200°C. **(d)** 300°C. The vibrational modes J_1_ and J_2_ representing 1T-MoS_2_ phase are evolving and increasing their intensities with increasing temperature, and conversely the typical vibrational mode E^1^_2g_ of 2H-MoS_2_ phase is diminishing with increasing temperature and dwell times.

**
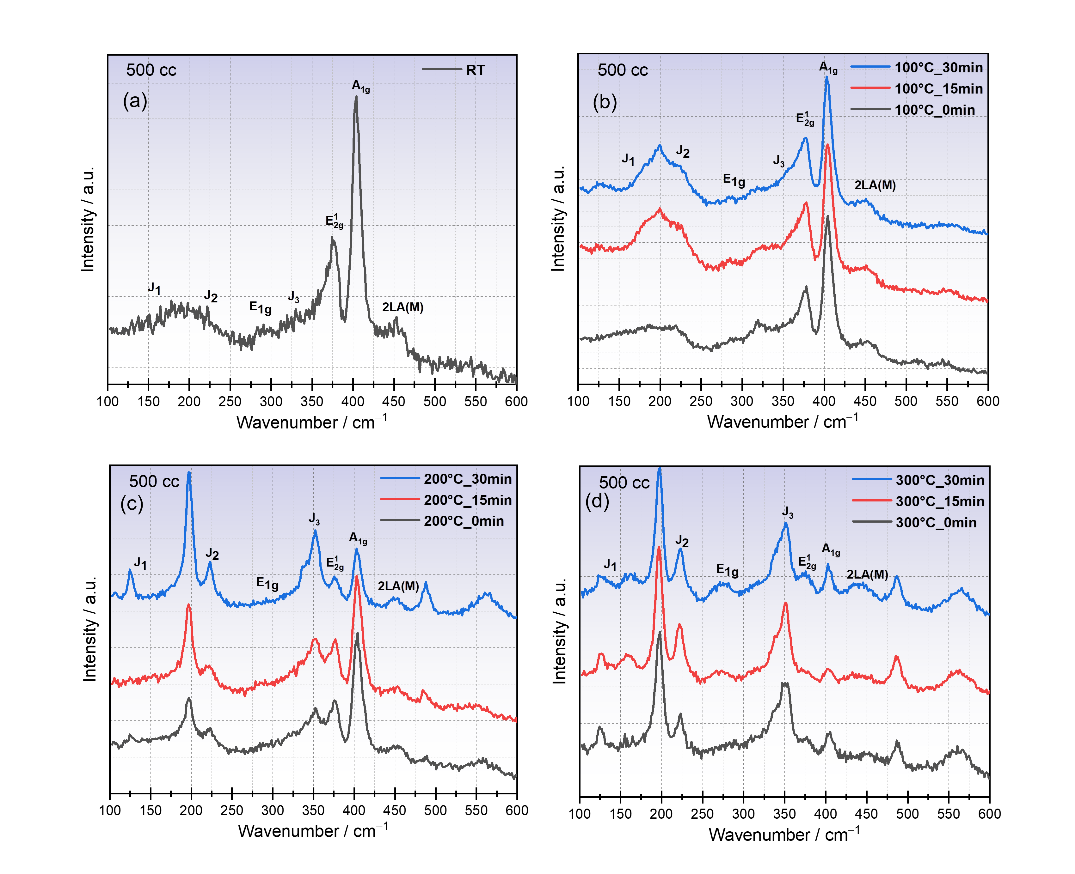
**

**Fig. S****3** In situ Raman heating for MSQD_500_ sample recorded at **(a)** RT. **(b)** 100°C. **(c)** 200°C. **(d)** 300°C. The vibrational modes J_1_ and J_2_ representing 1T-MoS_2_ phase are evolving and increasing their intensities with increasing temperature, and conversely the typical vibrational mode E^1^_2g_ and A_1g_ of 2H-MoS_2_ phase are diminishing with increasing temperature and dwell times.


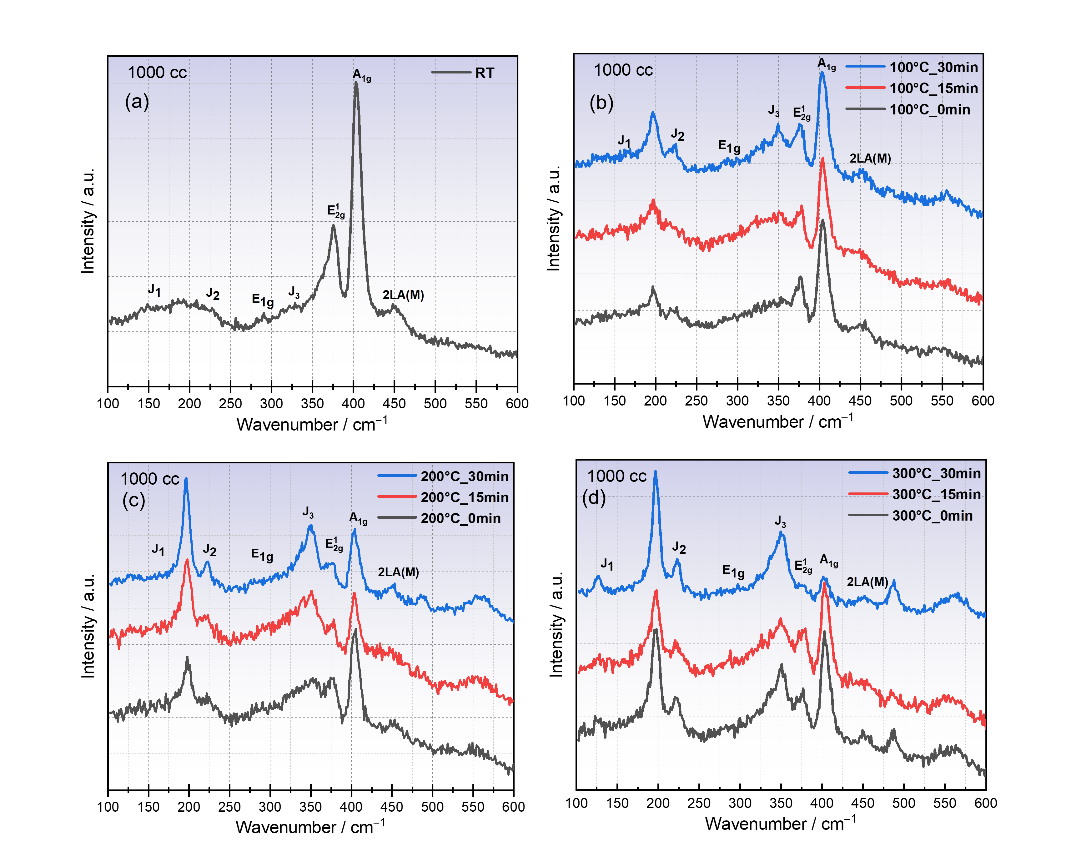


**Fig. S4** In situ Raman heating for MSQD_1000_ sample recorded at **(a)** RT. **(b)** 100°C. **(c)** 200°C. **(d)** 300°C. The vibrational modes J_1_ and J_2_ representing 1T-MoS_2_ phase are evolving and increasing their intensities with increasing temperature, and conversely the typical vibrational mode E^1^_2g_ of 2H-MoS_2_ phase is diminishing with increasing temperature and dwell times.

**
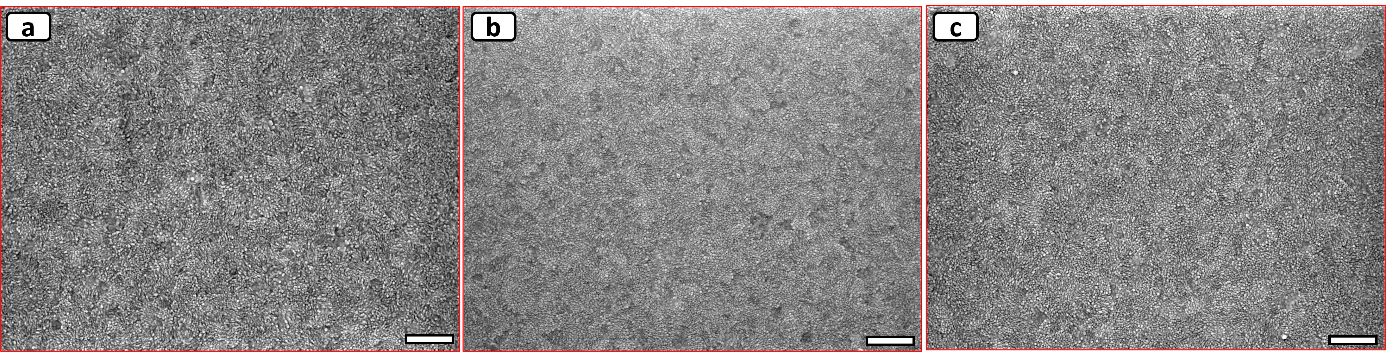
**

**Fig. S5** Microstructural analysis. SEM micrographs at low magnification (scale bar = 400 nm) for. **(a)** MSQD_250_. **(b)** MSQD_500_. **(c)** MSQD_1000_. For all samples, QDs are evenly distributed on the substrate surface.

**
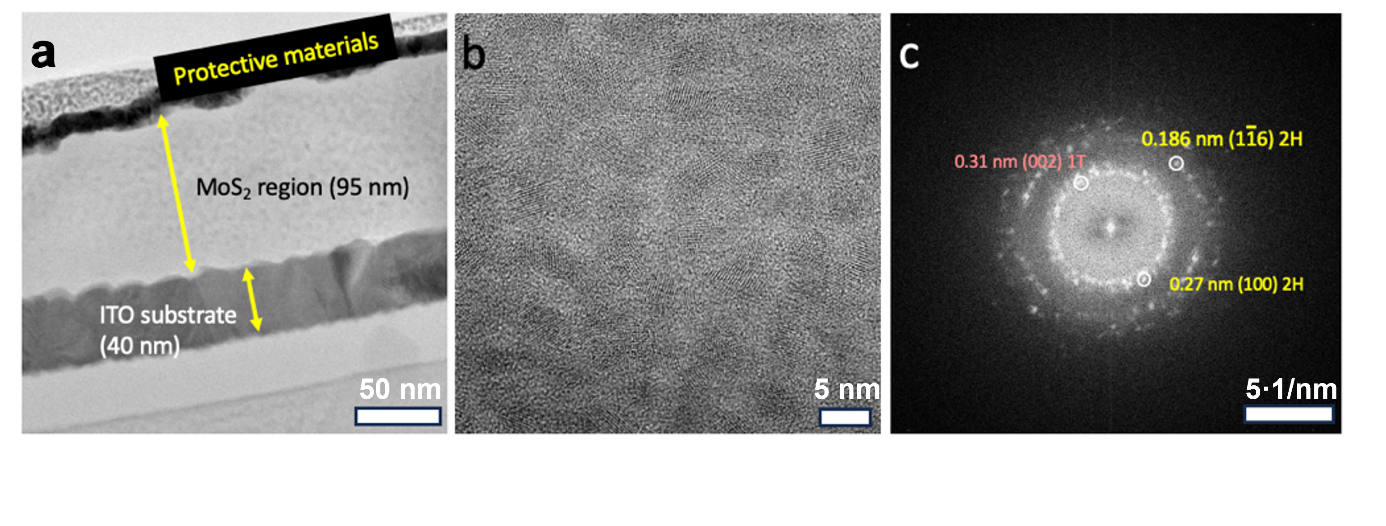
**

**Fig. S6** TEM analysis of MSQD_250_ sample. **(a)** Cross-sectional bright field TEM image showing different layers. **(b)** HRTEM image showing the nanocrystals of MoS_2_ embedded in amorphous matrix. **(c)** Fast Fourier transform (FFT) of image (b) showing the mixed phases 1T/2H-MoS_2_ crystals.

**
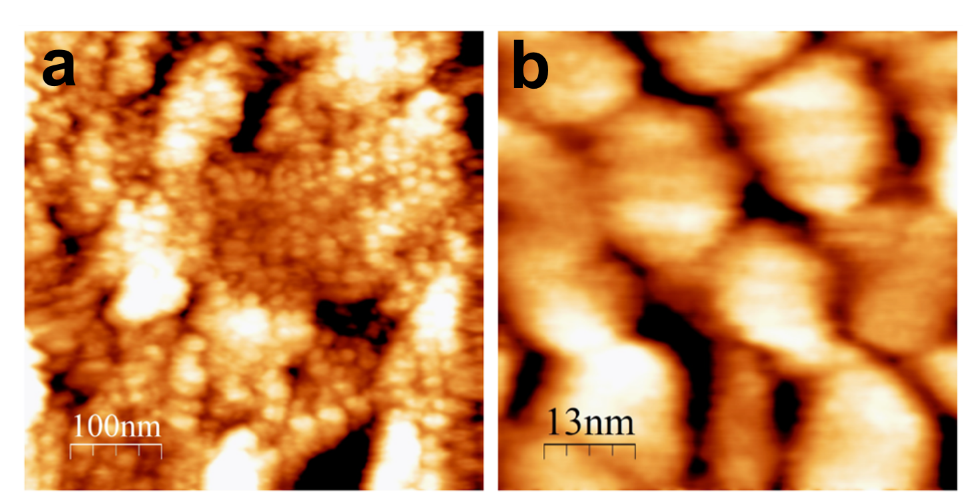
**

**Fig. S7** STM images of MSQD_500_ sample. **(a)** Low. **(b)** High magnification, mean QDs size in 12-15 nm range.


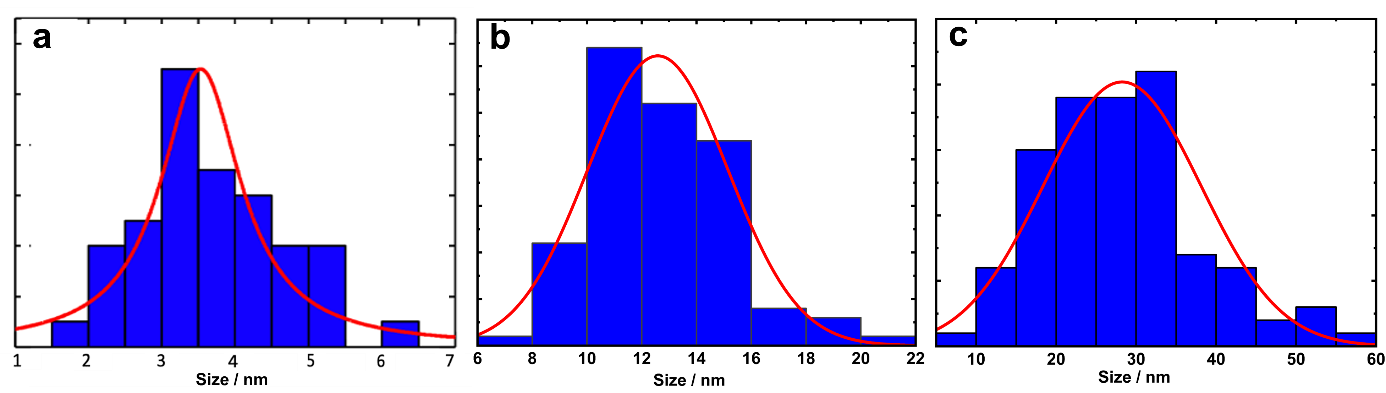


**Fig. S8** Size distribution. **(a)** MSQD_250_ with a mean nanocrystal size of 3.5 nm. **(b)** MSQD_500_ with a mean nanocrystal size of 12.6 nm. **(c)** MSQD_1000_ with a mean nanocrystal size of 28.4 nm.

**
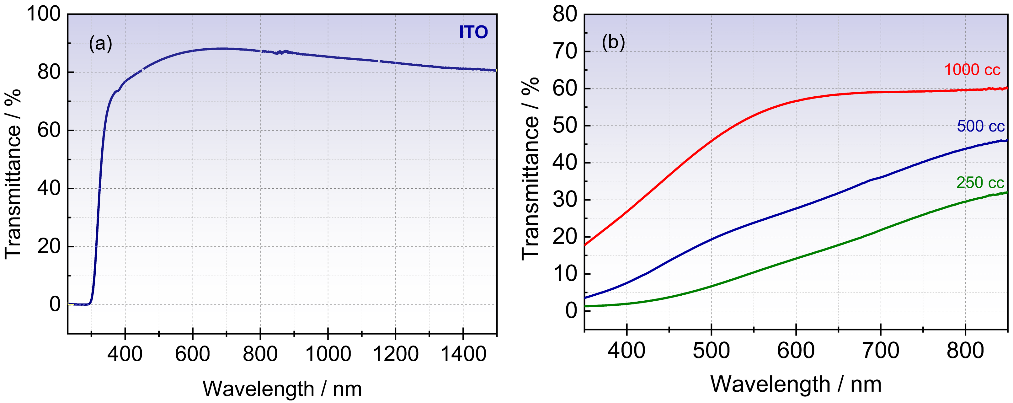
**

**Fig. S9** Optical transmittance. **(a)** Indium tin oxide coated glass substrate In_2_O_3_/SnO_2_. **(b)** MSQD_250_, MSQD_500_ and MSQD_1000_ samples.


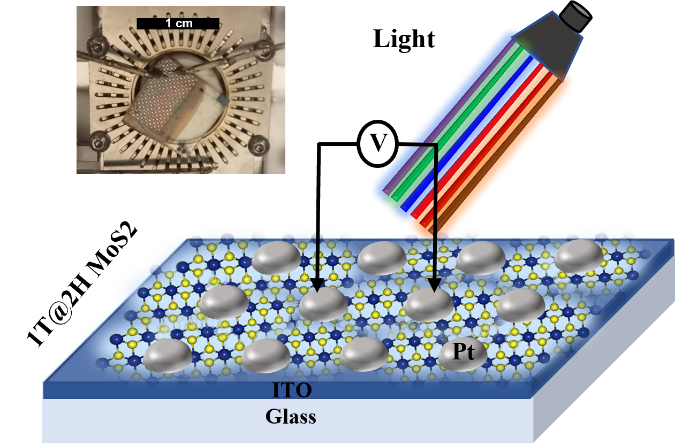


**Fig. S10** Schematic architecture of the tested device and its digital photograph.


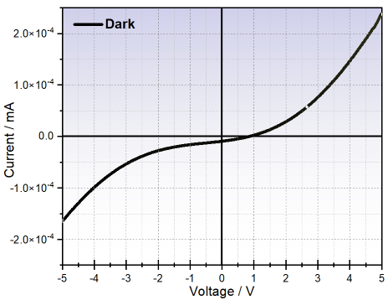


**Fig. S11** Measured dark current after 24 hours. Note the minimal value of dark current 0.2 µA.

**
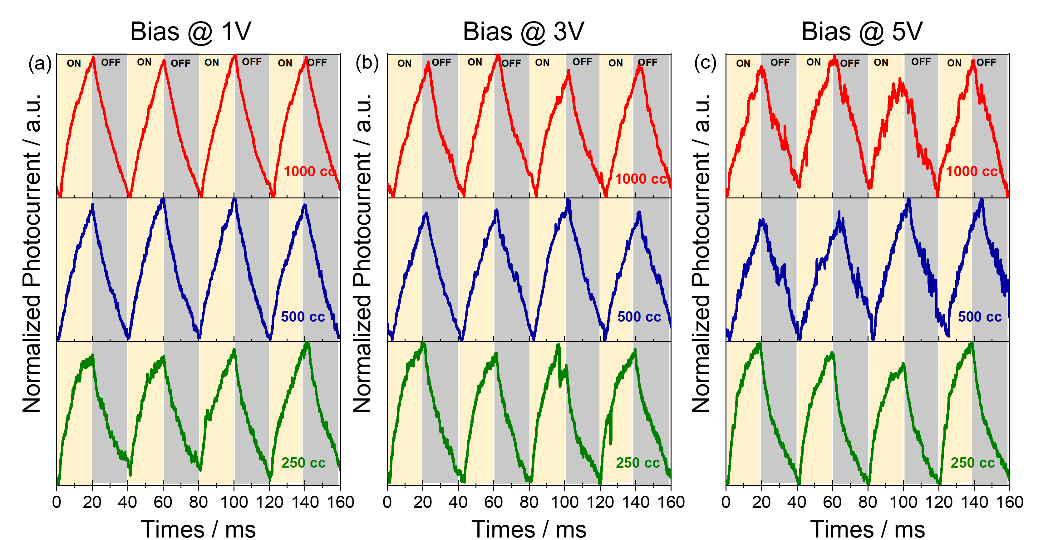
**

**Fig. S12** Chronoamperometry experiments and light on/off cycles (every 20 ms) carried out on MSQD_250_ (green), MSQD_500_ (blue) and MSQD_1000_ (red) under standard solar simulation with a *P*_light_= 100 mW$\text{·}$cm^–2^ at bias of. (**a)** 1 V. **(b)** 3 V. **(c)** 5V.

**
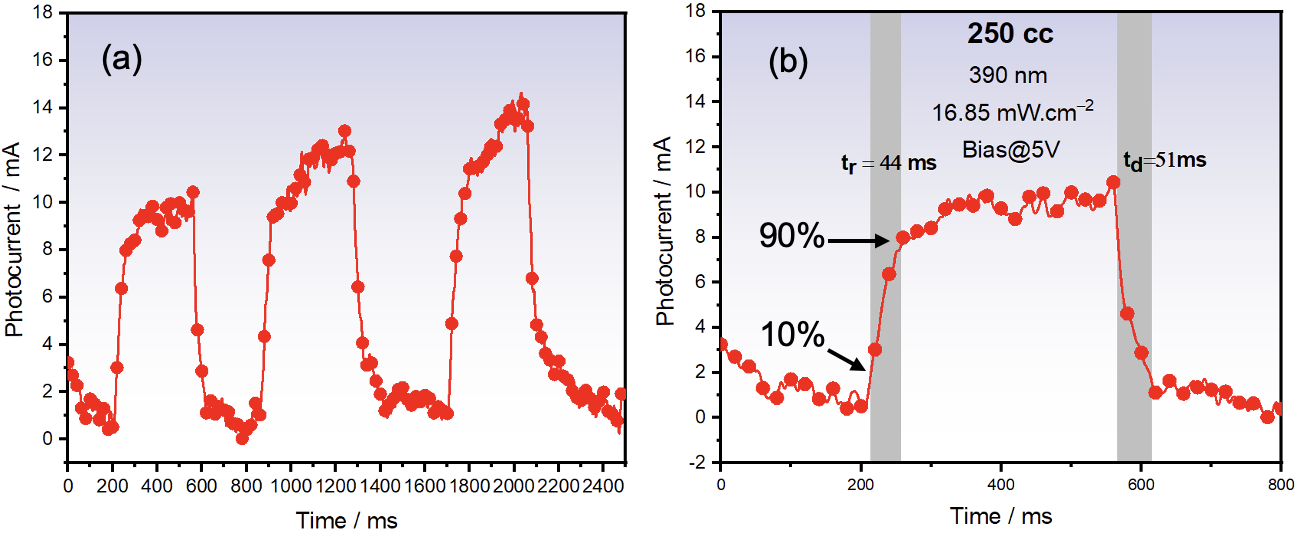
**

**Fig. S13** Chronoamperimetry experiments and light on/off for MSQD_250_ sample under 390 nm excitation, *P*_light_=16.85 mW$\text{·}$cm^–2^ and 5 V bias. (a) Actual photoresponse dynamics. (b) First cycle showing rise time t_r_ = 44ms and decay time t_d_ =51 ms.

**
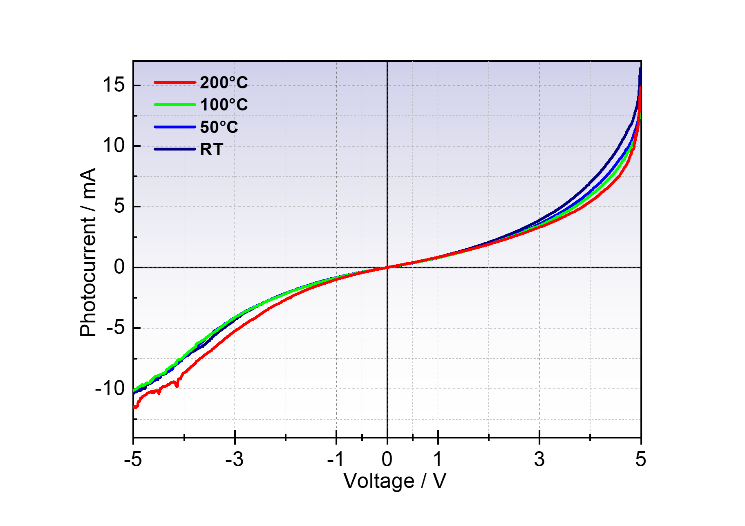
**

**Fig. S14** Photocurrent recorded under standard solar simulator at *P*_light_ =100 mW·cm^−2^ for MSQD_250_ at room temperature, 50°C, 100°C and 200°C.

**Table S1** Ratio of 1T-MoS_2_ with respect to 2H-MoS_2_ for all fabricated samples.

| Sample | 1T/2H-MoS2 ratio | |
| --- | --- | --- |
| MSQS_250_ MSQD_500_  MSQD_1000_ | | 0.74  1.38  2.35 |

**Table S2** Ratio of Mo with respect to S for all fabricated samples.

| Sample | Mo (At%) | S (At%) | Mo/S |
| --- | --- | --- | --- |
| MSQS_250_ MSQD_500_  MSQD_1000_ | 32.56  29.51  28.21 | 67.44  70.49  71.79 | 0.48  0.42  0.39 |

**Table S3** Optimized conditions for best photodetection performances for all samples obtained under solar simulator illumination at 5 V bias.

| Sample | *P*_ligh_ (mW$\text{·}$cm^–2^) | *Iph* (mA) | R (A$\text{·}$W^–1^) | D* (Jones) |
| --- | --- | --- | --- | --- |
| MSQS_250_ MSQD_500_  MSQD_1000_ | 40  60  100 | 15.15  5.28  4.25 | 758  176  85 | 5.2$\text{·}$10^13^  1.2$\text{·}$10^13^  5.8$\text{·}$10^12^ |
